# Elimination of hepatocyte‐derived DPP4 downregulates cardiac immune‐ and collagen‐related genes but does not alter cardiac function in aged male mice

**DOI:** 10.14814/phy2.70453

**Published:** 2025-08-07

**Authors:** Natasha A. Trzaskalski, Melissa M. Dann, Antonio Hanson, Branka Vulesevic, Evgenia Fadzeyeva, Natasha Jeraj, Ilka Lorenzen‐Schmidt, Rick Seymour, Erin E. Mulvihill

**Affiliations:** ^1^ Department of Biochemistry, Microbiology and Immunology, Faculty of Medicine The University of Ottawa Ottawa Ontario Canada; ^2^ The University of Ottawa Heart Institute Ottawa Ontario Canada; ^3^ Interdisciplinary School of Health Sciences University of Ottawa Ottawa Ontario Canada

**Keywords:** cardiovascular dysfunction, diastolic dysfunction, dipeptidyl peptidase‐4, echocardiography, hepatokine

## Abstract

The association between metabolic dysfunction–associated steatotic liver disease (MASLD) and cardiovascular disease is well characterized; however, the underlying mechanism is incompletely understood. Interestingly, hepatocyte‐specific silencing of dipeptidyl peptidase 4 (DPP4) prevents liver fibrosis and adipose tissue inflammation; however, how this affects the heart remains to be investigated. This study evaluates how diastolic function and molecular signatures of heart failure, like inflammation and fibrosis, are affected in male *Dpp4+/+*, *Dpp4−/−*, and *Dpp4flox/flox* mice injected with a TBG‐CRE to selectively eliminate DPP4 from hepatocytes (*Dpp4hep−/−*) and respective controls (*Dpp4GFP*), aged and fed an HFHC diet for 24 weeks. Mice underwent pulsed‐wave and tissue Doppler echocardiography. Further, speckle‐tracking strain analysis was performed to detect diastolic dysfunction. Differential mRNA analysis using the NanoString platform and qRT‐PCR were conducted using ventricular tissue to assess immunological pathway expression, as well as hypertrophy, modeling‐related, senescence, and metabolism gene expression. Immunological pathway analysis of ventricular tissue revealed downregulation of 12 immune‐related pathways in *Dpp4hep−/−* mice, including apoptosis and chemokine and cytokine signaling; however, this was not observed in *Dpp4−/−* mice. Further, fibrosis and ECM modeling‐related genes, *Col1a1*, *Col3a1*, *Ctgf*, and *Myh7*, were significantly upregulated in *Dpp4−/−* mice but unchanged in *Dpp4hep−/−* mice, compared to controls. Interestingly, cardiac hypertrophy and systolic and diastolic function evaluated with echocardiography were unchanged. Immune‐related pathways are downregulated in *Dpp4hep−/−* mice, while fibrosis genes are significantly upregulated in *Dpp4−/−* mice, compared to respective controls. Despite these molecular changes, cardiac hypertrophy and systolic and diastolic function were unchanged with systemic and organ‐specific loss of *Dpp4*.

## 1 INTRODUCTION

Currently, one in five Canadians is living with metabolic syndrome, a cluster of risk factors which increase the risk of cardiovascular disease (CVD) and type 2 diabetes (T2D) in adults (Sarafidis & Nilsson, 2006). Metabolic dysfunction‐associated steatotic liver disease (MASLD) is the most recent acronym introduced to appropriately describe hepatic lipid accumulation with at least one of five cardiovascular risk factors, including obesity, hyperglycemia, and hypertriglyceridemia (Rinella et al., 2023). Interestingly, MASLD appears to have extrahepatic consequences which can contribute to CVD progression (Bonci et al., 2015; Van Wagner et al., 2015). In fact, CVD is the leading cause of death in individuals with fatty liver disease (Targher et al., 2010). These individuals are also predisposed to atherogenic dyslipidemia that contributes to increasing the risk of atherosclerosis and the development of unstable plaques (Sinn et al., 2017). Interestingly, growing evidence suggests that MASLD, independent of well‐established cardiovascular risk factors, can still contribute significantly to CVD (Cakir et al., 2012) and may be linked to the release of proinflammatory and profibrogenic factors.

The liver, adipose tissue, and skeletal muscle secrete proteins, respectively called hepatokines, adipokines, and myokines, which influence biological functions in both local and peripheral tissues, resulting in inter‐tissue crosstalk (Priest & Tontonoz, 2019; Ren et al., 2022). One such hepatokine, previously regarded as an adipokine, is dipeptidyl peptidase‐4 (DPP4). It selectively cleaves N‐terminal dipeptides from substrates, the most well‐characterized being the incretin hormones glucagon‐like peptide‐1 (GLP‐1) and glucose‐dependent insulinotropic polypeptide (GIP), which are quickly inactivated, preventing postprandial insulin secretion (Lambeir et al., 2003; Mentlein, 1999; Mentlein et al., 1993). In line with this, we recently revealed increased active GLP‐1 in the portal vein and reduced hepatic glucose production with genetic elimination of hepatocyte‐derived *Dpp4* (*Dpp4* ^*hep−/−*^), as determined by hyperinsulinemic‐euglycemic clamps performed in 12–16‐week‐old mice fed a high‐fat high‐cholesterol (HFHC) diet for 12–16 weeks (Trzaskalski et al., 2022).

Through hepatocyte‐targeted siRNA, Ghorpade et al. silenced hepatocyte *Dpp4* and demonstrated reduced adipose tissue inflammation and insulin resistance in obese mice (Ghorpade et al., 2018). In *Dpp4* ^*hep−/−*^ mice, Varin et al. showed decreased inflammation in the liver and adipose tissue, but did not affect glucose tolerance (Varin et al., 2019). Conversely, Baumeier et al. (2017) overexpressed hepatic *Dpp4*, which resulted in adipose tissue inflammation, hypercholesterolemia, hepatic steatosis, and insulin resistance. In the context of fatty liver disease, Gorgens et al. (2019) revealed that hepatocyte‐specific siRNA‐mediated *Dpp4* knockout reduced hepatic steatosis in *db/db* mice, while we have also recently shown reduced hepatic fibrosis, as evidenced by Picosirius red staining and mRNA expression of immunological pathways in *Dpp4* ^*hep−/−*^ mice (Trzaskalski et al., 2022).

However, despite consistent evidence for reducing inflammation in the liver and adipose tissue, the impact of hepatocyte‐derived DPP4 in cardiovascular disease progression remains to be evaluated. Here, we investigate the role of hepatic DPP4 in ventricular function and preventing cardiac inflammation and fibrosis in a diet‐induced model of hepatic steatosis.

## 2 METHODS

### 2.1 Animals

Animals were cared for in accordance with the Canadian Council on Animal Care (CCAC). All experimental procedures were approved under AUP#2909 and AUP#2029 by the University of Ottawa Animal Care. Male mice were housed under a 12‐h light/dark cycle and maintained on high‐fat, high‐cholesterol (HFHC; 42% kcal from fat, 42.7% kcal from carbohydrates, 15.2% kcal from protein, 0.2% weight from cholesterol: TD88137, Envigo) diet. Male, whole body *Dpp4* knockout (*Dpp4* ^*−*/*−*^) mice, on a C57BL6 background, have been described (Marguet et al., 2000; Mulvihill et al., 2017). To generate hepatocyte‐specific *Dpp4* knockout(^*−*/*−*^) mice, male *Dpp4* ^*flox*/*flox*^ adult mice were intravenously injected with 1.5 × 10^11^ genome copies per mouse of adeno‐associated virus (AAV)8.TBG.pi.egfp.wpre.bgh (AAV‐GFP; control virus, #105535‐AAV8, *Dpp4* ^*GFP*^) or AAV8.TBG.PI.CRE.rBG (AAV‐Cre; #107787‐AAV8, *Dpp4* ^*hep−*/*−*^) prior to the onset of HFHC diet feeding. AAV constructs were obtained from the University of Pennsylvania Vector Core Lab (gift from James M. Wilson, Perelman School of Medicine, University of Pennsylvania, Philadelphia, PA). To assess cardiac dysfunction, experiments were performed in 16–28‐week‐old mice fed an HFHC diet for 24 weeks prior to sacrifice, where tissue collection was performed at 40–52 weeks of age, as previously described (Trzaskalski et al., 2022).

### 2.2 NanoString mRNA analysis

NanoString (NanoString Technologies, Inc., Seattle WA) nCounter® Mouse Immunology Panel (catalogue # XT‐CSO‐MIM1‐12) was used, where 100 ng RNA was incubated with reporter and capture probes, consisting of 547 immunology‐related mouse genes and 14 internal reference controls, for 16 h at 65°C as described.

Raw gene expression data were analyzed using NanoString's software, nSolver v4.0.70, with the Advanced Analysis Module v2.0.115 with background subtraction. Genes with counts below a threshold of 20 were excluded from subsequent analysis. Data normalization was completed on background‐subtracted samples using both the internal positive controls and selected consistent housekeeping genes used across all analyses in ventricular tissue: Ribosomal L19 (*Rpl19*), Peptidylprolyl isomerase A (*Ppia*), Eukaryotic translation elongation factor 1 gamma (*Eef1g*), Ornithine decarboxylase antizyme 1 (*Oaz1*), Glucuronidase beta (*Gusb*), TATA‐box binding protein (*Tbp*), Glucose‐6‐phosphate 1‐dehydrogenase X‐linked (*G6pdx*), Tubulin beta‐5 chainclass Ichain (*Tubb5*), DNA‐directed RNA polymerase II subunit RPB1ARPB1 (*Polr2a*), Succinate dehydrogenase complex flavoprotein subunit A (*Sdha*), Glyceraldehyde‐3‐phosphate dehydrogenase (*Gapdh*), and DNA‐directed RNA polymerase I subunit RPA2BRPA2 (*Polr1b*). Significant differential gene expression was determined through analyses using nSolver, which employs multivariate linear regression models to identify significant genes (mixture negative binomial, simplified negative binomial, or log‐linear model). Raw mRNA counts were log2 transformed, and significance was determined using a *T*‐test. Statistically significant, differentially expressed genes were identified as those with a *p* value <0.05. Ratios of log_2_ normalized transcript count data were generated for ventricular tissue from HFHC‐fed *Dpp4* ^*−/−*^ mice versus baseline HFHC‐fed WT mice and HFHC‐fed *Dpp4* ^*hep−/−*^ mice versus baseline HFHC‐fed *Dpp4* ^*GFP*^ mice.

Pathway scores generated from nSolver Advanced Analysis were standardized by Z‐scaling. ClustVis was used to perform supervised hierarchical clustering analysis and principal components analysis of log_2_‐transformed transcript count data and Z‐scaled pathway scores.

### 2.3 Plasma analysis

Analysis for plasma alanine aminotransferase (ALT), aspartate aminotransferase (AST), alkaline phosphatase (ALP), triglycerides (TG), cholesterol (C), low‐density lipoprotein (LDL)‐C, and high‐density lipoprotein (HDL)‐C were performed by the Pathology Core at Toronto Centre for Phenogenomics. The Beckman Coulter AU480 clinical chemistry analyzer was used in combination with appropriate reagents (ALT, AST, TG, cholesterol, LDL, and HDL), calibrators (Beckman Coulter Lyophilized Chemistry Calibrator Levels 1 and 2), and quality control materials (Bio‐Rad Liquid Assayed Multiqual 1 and 3).

### 2.4 RNA isolation, cDNA, and qRT‐PCR

Flash‐frozen mouse hearts, without atria, were powdered and homogenized with TRIzol™ Reagent (Invitrogen, 15596026) using a TissueLyser II system (Qiagen), and total RNA was extracted using the manufacturer's protocol. Reverse transcription was performed with a High‐Capacity cDNA Reverse Transcription Kit (Applied Biosystems™, 4368814). cDNA was subsequently used to assess mRNA expression by qRT‐PCR (QuantStudio 5 System, Thermo Fisher Scientific) with TaqMan Fast Advanced Master Mix (Thermo Fisher Scientific, 4444557) and TaqMan Gene Expression Assays (Thermo Fisher Scientific). The specific gene expression assays used are listed. Quantification of transcript levels was performed by the standard curve method, and expression levels for each gene were normalized to *Gapdh*.

**Table:** 

Oligonucleotides	Identifier
Collagen, type I, alpha 1 (*Col1a1*)	Mm00801666_g1
Collagen, type I3 alpha 1 (*Col3a1*)	Mm00802331_m1
Connective tissue growth factor (*Ctgf*)	Mm01192931_g1
Myosin, heavy polypeptide 7, cardiac muscle, beta (*Myh7*)	Mm00600555_m1
Natriuretic peptide type A (*Nppa*)	Mm01255747_g1
Natriuretic peptide type B (*Nppb*)	Mm01255770_g1
Matrix metallopeptidase 2 (Mmp2)	Mm00439498_m1
Matrix metallopeptidase 9 (*Mmp9*)	Mm01240562_g1
Discoidin domain receptor family, member 2 (*Ddr2*)	Mm00445614_m1
Vimentin (*Vim*)	Mm01333430_m1
Ankyrin repeat domain 1 (cardiac muscle) (*Ankrd1*)	Mm00496512_m1
Cyclin‐dependent kinase inhibitor 1A (P21) (Cdkn1a)	Mm00432448_m1
Patatin‐like phospholipase domain containing 2 (*Pnpla2*)	Mm00503040_m1
Acyl‐Coenzyme A dehydrogenase, medium chain (*Acadm*)	Mm01323360_g1
CD36 antigen (*Cd36*)	Mm00432403_m1
Peroxisome proliferative activated receptor, gamma, coactivator 1 alpha (*Pgc1α*)	Mm01208835_m1
Diacylglycerol O‐acyltransferase 1 (*Dgat1*)	Mm00515643_m1
Pyruvate dehydrogenase kinase, isoenzyme 4 (*Pdk4*)	Mm01166879_m1
Carnitine palmitoyltransferase 1b, muscle (*Cpt1b*)	Mm00487191_g1
Solute carrier family 27 (fatty acid transporter), member 1 (*Slc27a1*)	Mm00449511_m1

### 2.5 Echocardiography: Image acquisition

Echocardiographic images were acquired with the Vevo 3100 high‐frequency small animal ultrasound system (FUJIFILM VisualSonics, Toronto, Canada), using either the MX400 or MX550D linear array transducers. Mice were anesthetized with 1%–2% isoflurane in 2 L/min oxygen and positioned supine on a heated physiological monitoring platform to collect respiration and ECG traces. Body temperature was monitored using a rectal thermometer and maintained between 36°C and 38°C. Images of the left ventricle were taken at 16 weeks and 24 weeks after HFHC feeding, using parasternal long‐axis view in 2D brightness mode (B‐mode). To further assess the diastolic function of the left ventricle, pulsed wave and tissue Doppler were used to measure mitral valve inflow and mitral annulus velocity, respectively, in an apical four‐chamber view of the heart. E and A peaks were measured from 1 to 3 consecutive heartbeats between breaths.

### 2.6 Strain analysis

Echocardiographic images in long‐axis view were analyzed for longitudinal and radial strain and strain rate using Vevo Strain software (Visualsonics). Three consecutive cardiac cycles free of respiratory artifacts were analyzed. Epi‐ and endocardium of the heart were manually traced at the time of end‐diastole in the first cycle and adjusted to ensure accurate tracking of the epi‐ and endocardial borders throughout the three cycles. The software determined an average time course of strain and strain rate at 49 evenly spaced points along the perimeter of the epi‐ and endocardium. Since it is difficult for technical reasons to detect strains accurately in the apical region, the apex was excluded from the analysis, leaving 34 points along the anterior and posterior myocardium, as previously described (Trzaskalski et al., 2022). The average strain and strain rate time course was calculated from these points in each of the directions (longitudinal and radial) and the appropriate maxima/minima were chosen to represent systolic and diastolic strain and strain rate, respectively.

### 2.7 Statistics

All data were plotted and statistical analysis was performed using GraphPad Prism (version 9.5.1). Data are presented as the means ± SEM, analyzed by unpaired Student's *t*‐test with Welch's correction, between genetic knockouts and respective controls (*Dpp4* ^*+/+*^ vs. *Dpp4* ^*−/−*^ mice, and *Dpp4* ^*GFP*^ vs. *Dpp4* ^*hep−/−*^ mice). Statistical differences between groups were evaluated by *p <* 0.05, which was considered statistically significant.

## 3 RESULTS

### 3.1 In ventricular tissue, immune‐related genes and pathways are largely downregulated in HFHC‐fed Dpp4^hep−/−^ mice

**FIGURE 1:**
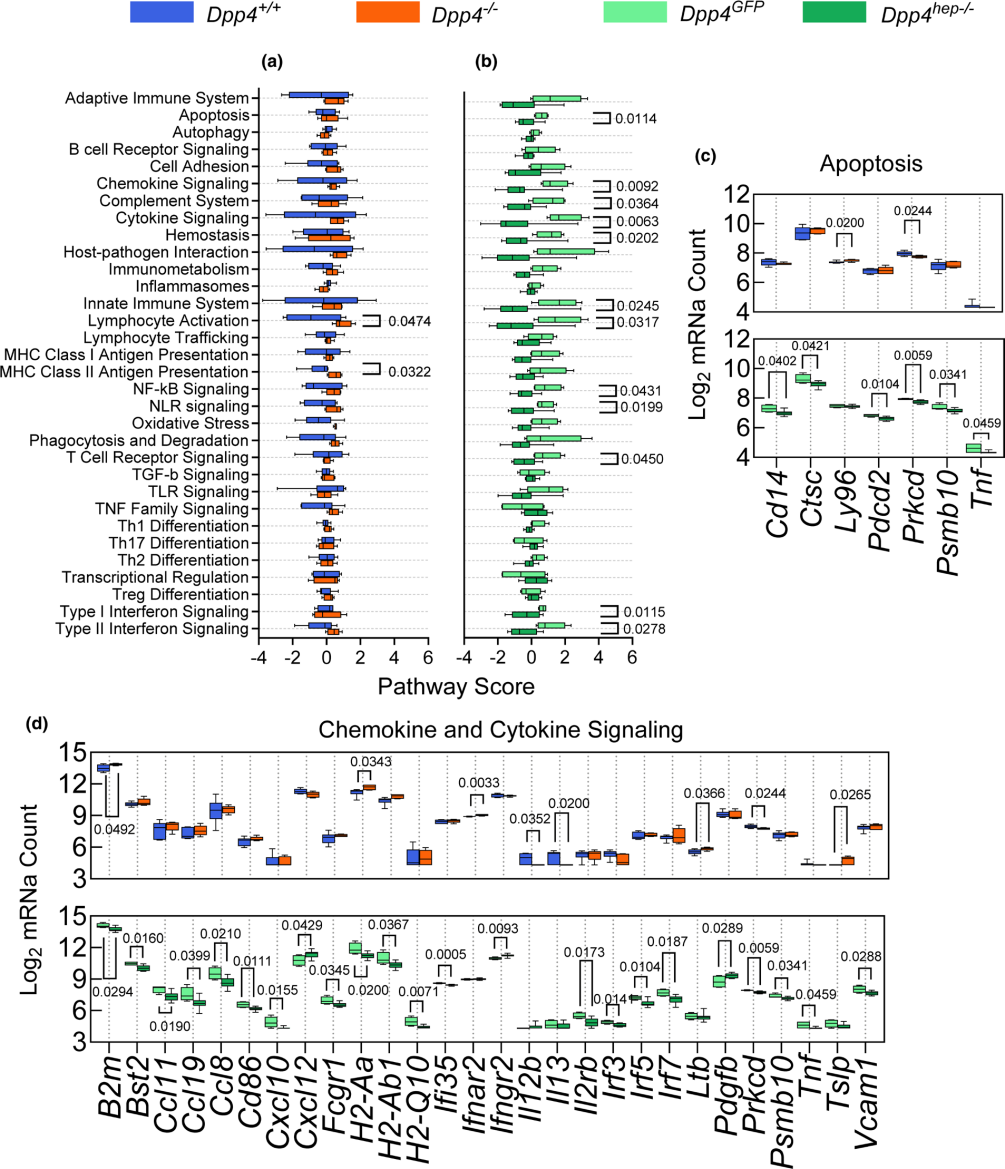
In *Dpp4* ^*hep−/−*^ mice, immune‐related genes and pathways are downregulated compared to controls, but relatively unchanged in *Dpp4* ^*−/−*^ mice. Scores of immunological pathways in ventricular tissue of (a) HFHC‐fed *Dpp4* ^*+/+*^ and *Dpp4* ^*−/−*^ mice and (b) HFHC‐fed *Dpp4* ^*GFP*^ and *Dpp4* ^*hep−/−*^ mice. Log_2_ normalized mRNA counts of genes associated with (c) apoptosis and (d) chemokine and cytokine signaling. Box and whisker plots: Box extends from the 25th to 75th percentiles, the whiskers go down to the smallest value and up to the largest. Data are presented as the means ± SEM, analyzed by unpaired students *t*‐test with Welch's correction, ns *p* < 0.05.

Plasma biochemical analysis revealed no significant changes between *Dpp4* ^*GFP*^ and *Dpp4* ^*hep−/−*^ mice; however, triglycerides and glucose were significantly decreased in *Dpp4* ^*−/−*^ mice compared to *Dpp4* ^*+/+*^ mice (Table 1) (Trzaskalski et al., 2022). Further, log_2_ normalized mRNA counts were published previously (Trzaskalski et al., 2022). Plasma and liver DPP4 activity tended to be decreased in *Dpp4* ^*−/−*^ and *Dpp4* ^*hep−/−*^ mice (Table 1) (Trzaskalski et al., 2022). Pathway analysis of gene expression in ventricular tissue (Figure 1a) and previously in liver tissue (Trzaskalski et al., 2022) of mice fed an HFHC diet for 24 weeks revealed distinct and opposing patterns across the *Dpp4* ^*+/+*^, *Dpp4* ^*−/−*^, *Dpp4* ^*hep−/−*^, and *Dpp4* ^*GFP*^ models. Using this experimental design, a MASLD phenotype was achieved (Trzaskalski et al., 2022). Pathways involved in MASLD pathogenesis were also significantly upregulated in *Dpp4* ^*−/−*^ mice, including apoptosis, immunometabolism, oxidative stress, and toll‐like receptor (TLR) signaling (via dysbiosis), which were attenuated in *Dpp4* ^*hep−/−*^ mice compared to *Dpp4* ^*GFP*^ mice (Trzaskalski et al., 2022). In ventricular tissue, however, only two pathways were significantly upregulated in *Dpp4* ^*−/−*^ mice compared to *Dpp4* ^*+/+*^ controls: lymphocyte activation and major histocompatibility complex (MHC) class II antigen presentation (Figure 1a). In contrast, 12 pathways were significantly downregulated in *Dpp4* ^*hep−/−*^ mice compared to their respective controls, *Dpp4* ^*GFP*^ mice (Figure 1b). This includes chemokine and cytokine signaling, apoptosis, and nuclear factor kappa B (NF‐κB) signaling. In addition, adaptive immune system, immunometabolism, oxidative stress, and T helper 2 (Th2) differentiation pathways were all trending downwards in *Dpp4* ^*hep−/−*^ mice compared to their respective controls, *Dpp4* ^*GFP*^ mice (Figure 1b). Further, individual apoptosis‐related genes were either unchanged or upregulated in *Dpp4* ^*−/−*^ mice compared to *Dpp4* ^*+/+*^ controls, including lymphocyte antigen 96 (*Ly96*) (Figure 1c). In *Dpp4* ^*hep−/−*^ mice compared to *Dpp4* ^*GFP*^ mice, almost all genes were significantly downregulated, including cluster of differentiation 14 (*Cd14*) and tumor necrosis factor (*Tnf*) (Figure 1c). This pattern was maintained when we looked at chemokine and cytokine signaling (Figure 1d). Most genes were significantly differentially downregulated in *Dpp4* ^*hep−/−*^ mice compared to their respective controls, *Dpp4* ^*GFP*^ mice, and these same genes were either unchanged or upregulated in *Dpp4* ^*−/−*^ mice compared to *Dpp4* ^*+/+*^ controls.

**TABLE 1:** Biochemical analysis of liver tissue and plasma of aged HFHC‐fed *Dpp4* ^*+/+*^, *Dpp4* ^*−/−*^, *Dpp4* ^*GFP*^, and *Dpp4* ^*hep−/−*^ mice.

	*Dpp4* ^*+/+*^	*Dpp4* ^*−/−*^	*p* Value	*Dpp4* ^*GFP*^	*Dpp4* ^*hep−/−*^	*p* Value
ALT (IU/L)	244.66 ± 269.34	128.26 ± 86.51	0.33	194.125 ± 117.70	325.85 ± 229.70	0.30
AST (IU/L)	202.21 ± 131.78	180.40 ± 34.31	0.70	224.675 ± 125.25	273.13 ± 86.66	0.38
ALP (IU/L)	26.95 ± 21.92	31.35 ± 39.65	0.79	41.15 ± 55.43	27.64 ± 22.80	0.45
Cholesterol (mg/dL)	293.60 ± 85.43	249.66 ± 100.21	0.38	261.35 ± 87.53	320.01 ± 50.18	0.09
HDL (mg/dL)	113.52 ± 22.89	102.71 ± 26.62	0.42	109.20 ± 31.28	126.55 ± 13.77	0.33
TG (mg/dL)	82.82 ± 21.06	56.95 ± 1.94	**0.01**	64.87 ± 3.37	66.32 ± 10.90	0.85
LDL (mg/dL)	34.71 ± 14.44	21.64 ± 10.48	0.08	23.26 ± 10.38	29.23 ± 8.92	0.19
Glucose (mg/dL)	373.88 ± 49.31	314.58 ± 79.22	0.10	290.45 ± 28.76	335.35 ± 87.56	0.16
Body weight (g)	49.07 ± 9.04	52.71 ± 12.10	0.40	49.49 ± 6.35	54.39 ± 7.26	0.25
Plasma DPP4 activity (nmol/min/mL)	6.60 ± 3.80	0.00 ± 0.00	**0.002**	7.67 ± 2.42	2.63 ± 0.49	**<0.0001**
Liver DPP4 activity (nmol/min/mg protein)	0.73 ± 0.72	0.00 ± 0.00	**0.045**	0.47 ± 0.30	0.08 ± 0.13	**0.002**

*Note*: Data are presented as the means ± SEM, analyzed by unpaired students *t*‐test with Welch's correction, between genetic knockouts and respective controls (*Dpp4* ^*+/+*^ vs. *Dpp4* ^*−/−*^ and *Dpp4* ^*GFP*^ vs. *Dpp4* ^*hep−/−*^), ns *p* < 0.05. Bold indicates Significance at *p* < 0.05.

### 3.2 Fibrosis‐related genes, but not hypertrophy or metabolism‐related genes, are upregulated in Dpp4^−/−^ mice and unchanged in Dpp4^hep−/−^ mice

**FIGURE 2:**
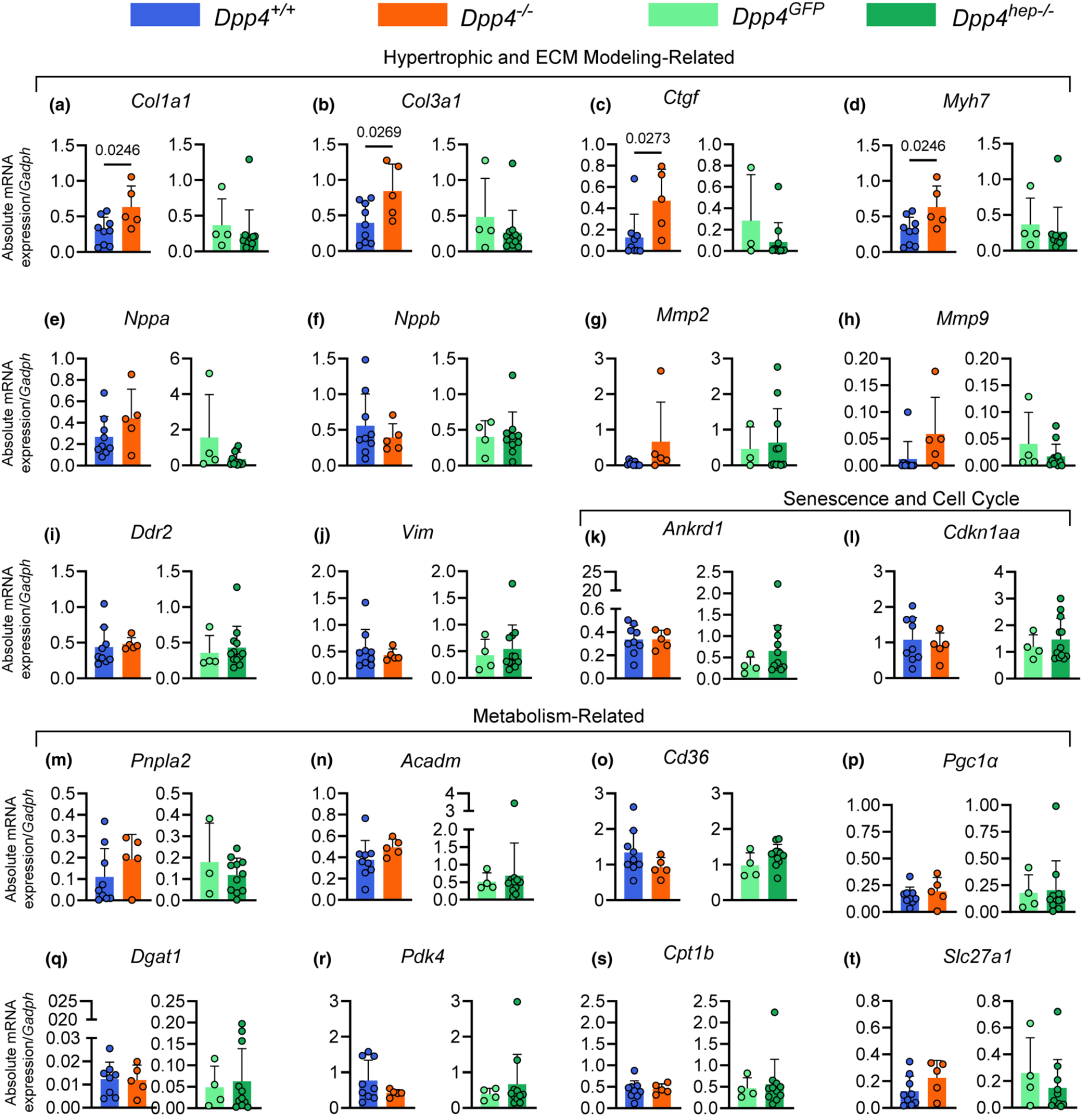
Fibrosis‐related genes are upregulated in *Dpp4* ^*−/−*^ mice and unchanged in *Dpp4* ^*hep−/−*^ mice, but no differences were detected in senescence and metabolism‐related genes. Ventricular mRNA abundance (relative to *Gapdh*) of hypertrophic and ECM modeling‐related genes (a) *Col1a1*, (b) *Col3a1*, (c) *Ctgf*, (d) *Myh7*, (e) *Nppa*, (f) *Nppb*, (g) *Mmp2*, (h) *Mmp9*, (i) *Ddr2*, and (j) *Vim*. Senescence and cell cycle genes (k) *Ankrd1* and (l) *Cdkn1a*. Metabolism‐related genes (m) *Pnpla2*, (n) *Acadm*, (o) *Cd36*, (p) *Pgc1α*, (q) *Dgat1*, (r) *Pdk4*, (s) *Cpt1b*, and (t) *Slc27a1*. Data are presented as the means ± SEM, analyzed by unpaired students *t*‐test with Welch's correction, ns *p* < 0.05.

Since immunological pathways and genes were downregulated in *Dpp4* ^*hep−/−*^ mice, we evaluated hypertrophy, fibrosis, and metabolism‐related genes using qRT‐PCR in ventricular tissue to determine if there were additional molecular changes. Interestingly, pro‐fibrotic genes collagen, type I, alpha 1(*Col1a1*) (Figure 2a), collagen, type 3, alpha 1 (*Col3a1*) (Figure 2b), connective tissue growth factor (Ctgf) (Figure 2c) and myosin, heavy polypeptide 7, cardiac muscle, beta (*Myh7*) (Figure 2d) were all significantly upregulated in *Dpp4* ^*−/−*^ mice compared to *Dpp4* ^*+/+*^ controls; however, they were unchanged in *Dpp4* ^*hep−/−*^ mice. Interestingly, additional hypertrophy and fibrosis‐related genes, including natriuretic peptide type A (*Nppa*) (Figure 2e), natriuretic peptide type A (*Nppb*) (Figure 2f), matrix metallopeptidase 2 (*Mmp2*) (Figure 2g), matrix metallopeptidase 9 (*Mmp9*) (Figure 2h), discoidin domain receptor family, member 2 (*Ddr2*) (Figure 2i) and vimentin (*Vim*) (Figure 2j) were all unchanged between groups. Similarly, senescence and cell cycle genes ankyrin repeat domain 1 (*Ankrd1*) (Figure 2k) and cyclin‐dependent kinase inhibitor 1A (*Cdkn1a*) (Figure 2l) were also unchanged. Following these trends, all metabolism‐related genes involved in fatty acid oxidation and glucose metabolism were unchanged between genotypes, including patatin‐like phospholipase domain containing 2 (*Pnpla2*) (Figure 2m), acyl‐Coenzyme A dehydrogenase, medium chain (*Acadm*) (Figure 2n), cluster of differentiation 14 (*Cd36*) (Figure 2o), peroxisome proliferative activated receptor, gamma, coactivator 1 alpha (*Pgc1a*) (Figure 2p), diacylglycerol O‐acyltransferase 1 (*Dgat1*) (Figure 2q), pyruvate dehydrogenase kinase, isoenzyme 4 (*Pdk4*) (Figure 2r), carnitine palmitoyltransferase 1b (*Cpt1b*) (Figure 2s), and solute carrier family 27 (fatty acid transporter), member 1 (*Slc27a1*) (Figure 2t).

### 3.3 Ventricular remodeling and systolic function are unchanged in Dpp4^−/−^ and Dpp4^hep−/−^ mice compared to respective controls

Immune and inflammation‐related genes and associated pathway analysis were significantly reduced in ventricular tissue from *Dpp4* ^*hep−/−*^ mice. Additionally, *Col1a1* and *Col3a1* were also induced in *Dpp4* ^*−/−*^ mice, prompting us to perform echocardiography to assess gross remodeling and assess if molecular changes translated to a functional cardiac phenotype. Heart weight normalized to total body weight was similar across genotypes and relatively low due to increased body weight and adiposity. However, while heart weight normalized to tibia length was also unchanged between groups, higher values compared to healthy mice indicate cardiac hypertrophy (Jia et al., 2018). Similarly, end diastolic and end systolic left ventricular mass were unchanged but increased compared to healthy mice in the literature (Gardin et al., 1995) (Table 2). Interventricular septal thickness at diastole was trending downwards in *Dpp4* ^*hep−/−*^ mice and unchanged in *Dpp4* ^*−/−*^ mice; however, at systole, there was no change but elevated compared to the literature (Jia et al., 2018). The left ventricular internal diameter (LVID) was elevated (Jia et al., 2018) but unchanged between genotypes at both diastole and systole, and the left ventricular posterior wall (LVPW) thickness was unchanged at diastole in both genotypes (Table 2). Interestingly, however, at systole, LVPW thickness significantly increased in *Dpp4* ^*−/−*^ mice, along with overall thickness, which was unchanged in *Dpp4* ^*hep−/−*^ mice. These structural changes and altered immune and fibrosis molecular signatures, however, did not translate to altered systolic function, as left ventricular ejection fraction (LVEF), stroke volume (SV), and cardiac output (CO) were all unchanged between genotypes but reduced overall (Table 2).

**TABLE 2:** Physiological and systolic echocardiographic analyses reveal no differences between genotypes and their controls.

		*Dpp4* ^*+/+*^	*Dpp4* ^*−/−*^	*p* Value	*Dpp4* ^*GFP*^	*Dpp4* ^*hep−/−*^	*p* Value
HW/BW	mg/g	3.69 ± 0.67	3.9 ± 1.03	0.64	3.86 ± 0.47	3.44 ± 0.64	0.25
HW/TL	mg/mm	7.88 ± 1.74	9.16 ± 1.58	0.19	7.78 ± 0.95	8.29 ± 0.99	0.38
EDLVM	mg	111 ± 19.97	106.4 ± 24.02	0.72	105.33 ± 19.22	92.77 ± 9.43	0.15
ESLVM	mg	106 ± 20.65	104 ± 26.83	0.88	102 ± 19.70	94.44 ± 14.10	0.48
IVS,d	mm	1.08 ± 0.12	1.18 ± 0.14	0.22	1.09 ± 0.17	0.93 ± 0.11	0.07
IVS,s	mm	1.53 ± 0.15	1.64 ± 0.18	0.21	1.54 ± 0.28	1.35 ± 0.17	0.15
LVID,d	mm	4.21 ± 0.41	4.01 ± 0.74	0.45	4.07 ± 0.29	4.24 ± 0.38	0.49
LVID,s	mm	2.92 ± 0.52	2.54 ± 0.71	0.21	2.84 ± 0.24	2.91 ± 0.42	0.79
LVPW,d	mm	0.92 ± 0.14	0.98 ± 0.17	0.42	0.92 ± 0.11	0.87 ± 0.13	0.57
LVPW,s	mm	1.24 ± 0.19	1.46 ± 0.17	***0.037***	1.27 ± 0.17	1.17 ± 0.17	0.37
Thickening	mm	0.31 ± 0.12	0.47 ± 0.05	***0.007***	0.35 ± 0.06	0.29 ± 0.07	0.25
HR	bpm	481.61 ± 48.48	482.14 ± 28.64	0.98	516.83 ± 28.80	456.81 ± 61.91	0.14
LVEF	%	56.32 ± 10.91	58.21 ± 10.04	0.73	49.41 ± 8.17	50.61 ± 9.32	0.82
SV	uL	36.7 ± 6.16	37.12 ± 9.47	0.92	33.8 ± 9.75	33.81 ± 5.94	0.10
CO	mL/min	17.43 ± 3.12	17.61 ± 4.10	0.93	17.53 ± 4.72	15.2 ± 2.72	0.24

*Note*: Data are presented as the means ± SE, analyzed by unpaired students *t*‐test with Welch's correction, between genetic knockouts and respective controls (*Dpp4* ^*+/+*^ vs. *Dpp4* ^*−/−*^ and *Dpp4* ^*GFP*^ vs. *Dpp4* ^*hep−/−*^), ns *p* < 0.05. Bold indicates significance at *p* < 0.05.

Abbreviations: bpm, beats per minute; BW, body weight; CO, cardiac output; d, diastole; EDLVM, end‐diastolic left ventricular mass; ESLVM, end‐systolic left ventricular mass; HR, heart rate; HW, heart weight; IVS, interventricular septal thickness; LVEF, ejection fraction; LVID, left ventricular internal diameter; LVPW, left ventricular posterior wall thickness; s, systole; SV, stroke volume; TL, tibia length.

### 3.4 Strain and strain rate are unaltered between genotypes

We used 2D strain analysis, a more sensitive measure of systolic function, to identify subclinical left ventricular dysfunction. Endocardial global longitudinal strain (GLS) was unchanged between genotypes; at aortic valve closure (AVC), radial strain (RS) was significantly increased in *Dpp4* ^*−/−*^ mice, while longitudinal strain (LS) was significantly decreased in *Dpp4* ^*hep−/−*^ mice (Table 3). However, these measurements were within a normal range as previously described (Gopal et al., 2021; Kalam et al., 2014). Consistently, radial strain rate at peak ejection (RSR at PE) was significantly increased in *Dpp4* ^*−/−*^ mice unchanged in *Dpp4* ^*hep−/−*^ mice and no further changes were observed in longitudinal strain rate at peak ejection (LSR at PE) in either group. At systole, time‐to‐peak (TTP) RS was trending upwards in *Dpp4* ^*hep−/−*^ mice but unchanged in *Dpp4* ^*−/−*^ mice (Table 3). Additional systolic measurements, including RS, TTP RSR, RSR, and TTP LS were all unchanged. LS, however, was trending downwards in *Dpp4* ^*hep−/−*^ mice and unchanged in *Dpp4* ^*−/−*^ mice, but TTP LSR and LSR were unchanged in both genotypes. When averaged across 34 segments along the myocardium of the left ventricle, average diastolic radial strain (aDRS) was significantly decreased in *Dpp4* ^*hep−/−*^ mice and unchanged in *Dpp4* ^*−/−*^ mice; however, average diastolic longitudinal strain (aDLS), average diastolic radial, and longitudinal strain rate were unchanged between genotypes (Table 3).

**TABLE 3:** Echocardiographic strain analysis in mice after 6 months of HFHC feeding reveals no differences between genotypes.

		*Dpp4* ^*+/+*^	*Dpp4* ^*−/−*^	*p* Value	*Dpp4* ^*GFP*^	*Dpp4* ^*hep−/−*^	*p* Value
GLS	%	−17.06 ± 5.09	−19.45 ± 3.14	0.37	−12.32 ± 5.64	−15.13 ± 3.38	0.31
RS at AVC	%	27.13 ± 7.83	38.38 ± 9.50	**0.048**	25.56 ± 4.93	25.71 ± 3.56	0.96
LS at AVC	%	−13.16 ± 6.51	−12.4 ± 8.43	0.86	−6.65 ± 5.93	−13.83 ± 3.93	**0.041**
RSR at PE	1/sec	5.88 ± 1.15	8.83 ± 2.42	**0.017**	6.84 ± 0.97	5.69 ± 1.34	0.21
LSR at PE	1/sec	−4.31 ± 2.12	−3.88 ± 2.05	0.74	−2.91 ± 0.93	−5.35 ± 2.20	0.10
TTP RS	ms	54.03 ± 5.39	57.2 ± 9.92	0.47	52.33 ± 3.83	60.83 ± 6.18	0.05
RS	%	31.77 ± 10.73	39.1 ± 9.29	0.24	27 ± 4.72	28.73 ± 4.97	0.61
TTP RSR	ms	24.62 ± 5.51	26 ± 6.35	0.69	29 ± 2.22	28.52 ± 7.62	0.92
RSR	1/sec	8.43 ± 2.35	9.6 ± 2.45	0.41	7.83 ± 0.14	7.53 ± 1.09	0.65
TTP LS	ms	55.09 ± 9.84	62.35 ± 22.11	0.43	61.04 ± 6.54	65.29 ± 12.91	0.60
LS	%	−14.58 ± 3.38	−14.06 ± 2.76	0.78	−11.05 ± 2.00	−14.36 ± 2.32	0.05
TTP LSR	ms	40.68 ± 9.81	50.72 ± 16.90	0.20	47.41 ± 3.39	57.25 ± 15.22	0.31
LSR	1/sec	−8.93 ± 1.93	−8.41 ± 2.00	0.65	−7.95 ± 0.56	−8.14 ± 1.57	0.85
aDRS	n/a	−0.8 ± 0.58	−0.71 ± 0.98	0.83	−0.29 ± 0.26	−1.6 ± 0.88	**0.034**
aDLS	n/a	1.1 ± 1.09	0.96 ± 1.80	0.84	0.64 ± 0.72	1.35 ± 0.93	0.26
aDRSR	1/sec	−10.27 ± 3.02	−12.99 ± 3.55	0.14	−9.08 ± 0.86	−10.58 ± 1.83	0.21
aDLSR	1/sec	4.81 ± 1.60	4.75 ± 2.25	0.96	3.92 ± 1.86	5.31 ± 2.49	0.40

*Note*: Data are presented as the means ± SE, analyzed by unpaired students *t*‐test with Welch's correction, between genetic knockouts and respective controls (*Dpp4* ^*+/+*^ vs. *Dpp4* ^*−/−*^ and *Dpp4* ^*GFP*^ vs. *Dpp4* ^*hep−/−*^), ns *p* < 0.05. Bold indicates significance at *p* < 0.05.

Abbreviations: a, average; AVC, aortic valve closure; DLS, diastolic longitudinal strain; DLSR, diastolic longitudinal strain rate; DRS, diastolic radial strain; DRSR, diastolic radial strain rate; GLS, global longitudinal strain; LS, longitudinal strain; LSR, longitudinal strain rate; n/a, not applicable; PE, peak ejection; RS, radial strain; RSR, radial strain rate; TTP, time‐to‐peak.

### 3.5 Pulsed wave and tissue Doppler echocardiography revealed no differences in Dpp4^−/−^ and Dpp4^hep−/−^ mice

In addition to strain analysis, we used pulsed wave and tissue Doppler echocardiography to assess diastolic function further. Isovolumetric contraction and relaxation times were unchanged between genotypes. In line with this, mitral valve atrial contraction (A) and rapid filling (E) velocities were unchanged between genotypes. Similarly, mitral annular velocity during atrial contraction (A') and rapid filling (E') were also unchanged between genotypes. Further, the resulting ratios were all unchanged between genotypes, including E'/A', E/A, and E/E'. However, there is a trend toward mild diastolic dysfunction in *Dpp4* ^*hep−/−*^ mice indicated by reduced E'/A' and E/A ratios compared to *Dpp4* ^*GFP*^ control mice (Table 4). Consistent with the literature, diastolic dysfunction was not evident in these mice (Wu et al., 2014).

**TABLE 4:** Echocardiographic pulsed wave Doppler and tissue Doppler analysis reveal no differences between genotypes after 6 months of HFHC feeding.

		*Dpp4* ^*+/+*^	*Dpp4* ^*−/−*^	*p* Value	*Dpp4* ^*GFP*^	*Dpp4* ^*hep−/−*^	*p* Value
A'	mm/s	−24.93 ± 7.14	−26.19 ± 6.37	0.75	−25.74 ± 6.61	−32.91 ± 8.27	0.15
E'	mm/s	−33.36 ± 12.83	−37.42 ± 9.10	0.54	−39.44 ± 16.17	−40.12 ± 13.58	0.94
IVCT	ms	13.97 ± 2.96	14.24 ± 3.18	0.88	13.77 ± 1.17	15.87 ± 3.50	0.27
IVRT	ms	13.42 ± 2.79	15.31 ± 2.77	0.24	11.89 ± 1.39	13.84 ± 2.68	0.20
MV A	mm/s	609.12 ± 107.18	692.14 ± 193.81	0.32	617.59 ± 169.12	645.53 ± 91.44	0.68
MV E	mm/s	781.97 ± 51.71	874.03 ± 130.21	0.07	811.13 ± 90.06	777.54 ± 102.02	0.57
E'/A'		1.35 ± 0.36	1.43 ± 0.19	0.65	1.52 ± 0.36	1.24 ± 0.33	0.18
MV E/A		1.31 ± 0.24	1.25 ± 0.25	0.68	1.38 ± 0.40	1.21 ± 0.15	0.21
MV E/E'		25.86 ± 7.72	24.19 ± 5.87	0.68	22.62 ± 6.89	21.09 ± 7.00	0.71
HR	bpm	519.1 ± 53.62	517.2 ± 37.36	0.95	574.75 ± 24.74	499.72 ± 49.34	**0.013**

*Note*: Data are presented as the means ± SEM, analyzed by unpaired students *t*‐test with Welch's correction, between genetic knockouts and respective controls (*Dpp4* ^*+/+*^ vs. *Dpp4* ^*−/−*^ and *Dpp4* ^*GFP*^ vs. *Dpp4* ^*hep−/−*^), ns *p* < 0.05. Bold indicates significance at *p* < 0.05.

Abbreviations: A', tissue doppler mitral valve A' wave; BPM, beats per minute; E', tissue doppler mitral valve E' wave; HR, heart rate; IVCT, isovolumetric contraction time; IVRT, isovolumetric relaxation time; MV A, mitral valve A wave; MV E, mitral valve E wave; n/a, not applicable.

## 4 DISCUSSION

Chronic inflammation and systemic disruption of metabolic homeostasis, such as that observed in obesity and MASLD are established precursors to diastolic heart failure (HF) (Shulman, 2014). Altered levels of several hepatokines like adropin (Chou et al., 2016), fetuin‐A, fibroblast growth factor 21 (FGF‐21), alpha‐1‐microglobulin, and selenoprotein P (Sell et al., 2013) have been implicated in the modulation of cardiac function via disturbances in inflammation, fibrosis, and adverse left ventricular (LV) remodeling. DPP4 has been previously established as a hepatokine; however, its role in CVD progression is incompletely defined. Circulating DPP4 levels are significantly increased in mice and humans in metabolic disease and obesity (Ghorpade et al., 2018; Lamers et al., 2011; Sell et al., 2013; Varin et al., 2019). Importantly, hepatocyte‐derived DPP4 has been identified as a pro‐inflammatory peptide, while regulation of GLP‐1 bioactivity and regulation of glucose has been ascribed to endothelial cell‐derived DPP4 (Varin et al., 2019). Chronic inflammation and systemic disruption of metabolic homeostasis, such as that observed in obesity and MASLD have established precursors to diastolic HF (Garg et al., 2021; Shulman, 2014). The present data demonstrate that the elimination of *Dpp4* specifically in hepatocytes in aged, male, HFHC‐fed mice significantly reduces immune‐related genes (i.e., *Tnf*, *Il12b*, and *Mcp‐2* (*Ccl8*)) and associated pathways in ventricular tissue compared to their controls, *Dpp4* ^*GFP*^. This is not observed in *Dpp4* ^*−/−*^ mice compared to *Dpp4* ^*+/+*^ mice, which is consistent with previous reports in euglycemic *Dpp4* ^*−/−*^ mice after trans‐aortic constriction (TAC) surgery, where markers of inflammation were significantly increased (Mulvihill et al., 2016). Similar to our findings, silencing hepatocyte‐specific *Dpp4* in diet‐induced obese (DIO) mice effectively suppressed visceral adipose tissue (VAT) inflammation identified by decreased *Il6*, *Mcp‐1*/*Ccl2*, *Tnfa*, and *Il1b* mRNA expression (Ghorpade et al., 2018). Our results are additionally supported by Varin et al. (2019) where mRNA expression of inflammatory cytokines (*Il1b*, *Il2*, *Il12b*, and *Mcp‐1*/*Ccl2*) was decreased in epidydimal white adipose tissue in HFHC‐fed *Dpp4* ^*hep−/−*^ mice. These results, collectively with our data, reveal a significant inflammatory role of hepatocyte‐derived DPP4 in peripheral tissue, namely, ventricular tissue.

Myocardial fibrosis and stiffening in diastolic HF are linked to increased mortality in clinical studies, making it an important therapeutic target (Garg et al., 2021). We recently revealed increased hepatic fibrosis in aged, HFHC‐fed *Dpp4* ^*−/−*^ mice, which was not observed in *Dpp4* ^*hep−/−*^ mice, suggesting an anti‐fibrotic role for DPP4, but not that derived from hepatocytes (Trzaskalski et al., 2022). Consistently, we report that hypertrophic and modeling‐related genes, *Col1a1*, *Col3a1*, *Ctgf*, and *Myh7* were significantly increased in *Dpp4* ^*−/−*^ mice compared to *Dpp4* ^*+/+*^; however, there was no change in *Dpp4* ^*hep−/−*^ mice compared to *Dpp4* ^*GFP*^ mice. Our findings are supported by others who use DPP4 inhibitors and systemic genetic elimination. Mulvihill et al. (2016) found that fibrosis was decreased in euglycemic *Dpp4* ^*−/−*^ mice; however, inducers and markers of fibrosis were elevated in aged, diabetic, high‐fat diet‐fed DPP4 inhibitor (MK‐0626) treated mice after TAC surgery. Further, in mice with angiotensin II‐induced chronic HF, DPP4 inhibitor, Teneligliptin, did not attenuate increases in mRNA expression of type 1 and 3 collagens (Okabe et al., 2020). In contrast, Shigeta et al. demonstrated that both systemic genetic elimination and inhibition with vildagliptin reversed interstitial fibrosis and decreased *Ctgf* mRNA levels caused by streptozotocin‐induced diabetes after TAC surgery in male Fischer 344 rats. In this same study, however, mRNA expression levels of collagen types 1 and 3, and *Mmp9* were DPP4 dependent; once *Dpp4* was systemically and genetically eliminated, these genes were significantly downregulated in cardiac tissue, most notably in diabetic mice. Similar to our findings, however, *Nppa* and *Nppb* expression was unaltered with the genetic elimination of *Dpp4* (Shigeta et al., 2012). Our results are additionally contested by studies that use pharmacological inhibition rather than genetic elimination, generally in younger, euglycemic rodents. In 4‐week‐old C57BL6/J mice fed a high‐fat/high‐fructose Western diet treated with MK‐0626 for 16 weeks, inhibition ameliorated diet‐induced cardiac fibrosis and oxidant stress (Bostick et al., 2014). Vildagliptin exerted a similar cardioprotective phenotype in C57BL6/J mice after TAC surgery; both myocardial apoptosis and fibrosis were attenuated (Takahashi et al., 2013). This improvement in fibrosis, or lack thereof, with pharmacological inhibition versus genetic elimination of *Dpp4*, may be a result of its enzymatic and nonenzymatic capacity. DPP4 is reported to cleave collagen (Ghersi et al., 2002); however, it has also been shown to bind to extracellular matrix (ECM) components, like fibronectin, and form complexes with fibroblast activation protein to degrade ECM (Ghersi et al., 2002, 2006; Ohm et al., 2022). Interestingly, linagliptin can block DPP4 and ECM component interactions, preventing signal transduction (Hasan & Hocher, 2017) and fibroblast activation (van Heerebeek et al., 2008; Lee et al., 2020). Systemic genetic elimination of *Dpp4* is hypothesized to prevent fibroblast activation and ECM degradation as previously described; this was not directly assessed in this study; however, we saw no changes in ECM remodeling‐related genes (*Mmp2*, *Mmp9*, *Ddr2*, and *Vim*). We also see no change in fibrosis in our *Dpp4* ^*hep−/−*^ mice; this may suggest that DPP4 from the liver, in aged, HFHC‐fed mice, may be pro‐fibrotic, and its genetic elimination is cardioprotective. However, a longer timeline may be required to see an impact on cardiac function.

Molecular changes like inflammation and fibrosis contribute to concentric hypertrophy, inadequate ventricular filling, and less blood perfusion to the periphery (Tadinada et al., 2021; Phan et al., 2012). In our study, we see only modest hypertrophy in our *Dpp4* ^*−/−*^ mice compared to *Dpp4* ^*+/+*^ mice, where both left ventricular posterior wall thickness and overall thickening are significantly increased, with no differences in cardiac function like LVEF. This is not observed in our *Dpp4* ^*hep−/−*^ mice, however. Consistent with our findings, Shigeta et al. (2012) reported significantly increased LV hypertrophy via posterior wall thickness in *Dpp4* ^*−/−*^ mice after TAC surgery compared to controls, while the cardiac function was unchanged. Similarly, Mulvihill et al. (2016) revealed that aged, diabetic mice treated with MK‐0626 possessed modest cardiac hypertrophy and impaired cardiac function.

## 5 LIMITATIONS

Left ventricular stiffness and inflexibility, preventing adequate relaxation during diastole, is prevalent in patients with T2D and typically precedes systolic dysfunction (van Heerebeek et al., 2008). We observed no improvements in diastolic function with systemic or hepatocyte‐specific *Dpp4* genetic elimination. The present study used HFHC feeding and age to facilitate cardiac dysfunction. However, this was not sufficient to induce a measurable phenotype, similar to previous studies that illustrate resilience among C57BL/6J mice; the background genotype of the mice used in this study developed liver disease rapidly on this diet (Eccleston et al., 2011; Savard et al., 2013). Despite extensive lard‐based HFD feeding and saturated fat‐rich diet feeding, cardiac dysfunction was not observed. Further, high‐fat feeding did result in cardiac hypertrophy; however, fibrosis and diastolic dysfunction were unchanged.

The use of diet‐induced HF does not indicate whether *Dpp4* genetic elimination could preserve cardiac function. Future studies could use a surgical intervention, such as trans‐aortic constriction, to induce pressure overload and uncover subtle functional changes otherwise not revealed in our less invasive protocol. Additionally, developmental compensation in congenital knockout models could influence phenotypic outcomes, whereas inducible knockouts may allow for temporal control that better isolates the effects of gene deletion in adulthood. These differences may contribute to some of the observed variability between models and highlight the importance of model selection when interpreting results. Further, this study was not designed to completely illuminate the nonenzymatic role that DPP4 may have in diastolic HF. Future studies could employ in vitro binding assays with isolated primary cardiomyocytes and inflammatory and fibrotic binding partners like adenosine deaminase (ADA), caveolin‐1, and fibrinogen.

Considering the lack of systolic and diastolic changes in our study, we could additionally employ stress echocardiography by administering dobutamine to mice. This has previously been identified as a technique in clinical and preclinical research that can reveal subtle changes in cardiac function which may otherwise remain hidden (Settelmeier et al., 2021). Interestingly, in patients with ischemic CVD and preserved LV function, a single dose of sitagliptin (100 mg) significantly improved LVEF during a stress echo with dobutamine at rest, at peak stress, and 30 min afterwards (Read et al., 2010).

## 6 CONCLUSION

In summary, our study demonstrates that hepatocyte‐derived *Dpp4* plays a distinct role in modulating immune response and fibrotic gene expression in cardiac tissue under conditions of metabolic stress. Hypertrophy and modeling‐related gene expression are significantly increased in *Dpp4* ^*−/−*^ HFHC‐fed mice; however, this is not observed in *Dpp4* ^*hep−/−*^ mice, compared to controls. Despite changes in inflammation and fibrosis, there were no changes in cardiac hypertrophy, systolic, and diastolic function.

## AUTHOR CONTRIBUTIONS

EEM: Conceived and designed research; NAT, MMD, AH, BV, EF, NJ, ILS, RS, and EEM: performed experiments; NAT, MMD, and BV: analyzed data; NAT, MMD, BV, and EEM: interpreted results of experiments; NAT: prepared figures; NAT, BV, and EEM: drafted manuscript; NAT and EEM: edited and revised manuscript; EEM: approved final version of manuscript.

## FUNDING INFORMATION

The Canadian Institutes of Health Research (CIHR) supported this work (ARJ‐162628, Project Grant 156136) and a Heart and Stroke National New Investigator award to EEM. NAT was supported by a UOHI endowment scholarship. BV was supported by a CIHR postdoctoral fellowship.

## CONFLICT OF INTEREST STATEMENT

The authors have nothing to declare in relation to this work.

## ETHICS STATEMENT

All procedres and methods used within this study were re‐viewed and approved by the University of Ottawa Animal Care Committee and in accordance with guidelines of the Canadian Council on Animal Care prior to commencment ofthe study.

## Data Availability

Data are available on request.
